# Appraising Hospital Performance by Using the JCHAO/CMS Quality Measures in Southern Italy

**DOI:** 10.1371/journal.pone.0048923

**Published:** 2012-11-07

**Authors:** Domenico Flotta, Paolo Rizza, Pierluigi Coscarelli, Claudia Pileggi, Carmelo G. A. Nobile, Maria Pavia

**Affiliations:** Department of Health Sciences, University of Catanzaro ‘Magna Græcia’, Catanzaro, Italy; S.G.Battista Hospital, Italy

## Abstract

**Objectives:**

The main objective of the present study was to estimate the uptake to quality indicators that reflect the current evidence-based recommendations and guidelines.

**Methods:**

A retrospective review of medical records of patients admitted to two hospitals in the South of Italy was conducted. For the purposes of the analysis, a sets of quality indicators has been used from the Joint Commission on Accreditation of Hospital Organizations and Centers for Medicare & Medicaid Services. Four areas of care were selected: acute myocardial infarction (AMI), heart failure (HF), pneumonia (PN), and surgical care improvement project (SCIP). Frequency or median was calculated, as appropriate, for each indicator. A composite score was calculated to estimate the overall performance for each area of care.

**Results:**

A total of 1772 medical records were reviewed. The adherence rates showed a wide-ranging variability among the selected indicators. The use of aspirin and angiotensin-converting enzyme inhibitor (ACEI) or angiotensin receptor blocker (ARB) for AMI, the use of ACEI or ARB for HF, the use of appropriate thromboembolism prophylaxis and appropriate hair removal for surgical patients almost approached optimal adherence. At the other extreme, rates regarding adherence to smoking-cessation counseling in AMI and HF patients, discharge instructions in HF patients, and influenza and pneumococcal vaccination in pneumonia patients were noticeably intangible. Overall, the recommended processes of care among eligible patients were provided in 70% for AMI, in 32.4% for HF, in 46.4% for PN, and in 46% for SCIP.

**Conclusions:**

The results show that there is still substantial work that lies ahead on the way to improve the uptake to evidence-based processes of care. Improvement initiatives should be focused more on domains of healthcare than on specific conditions, especially on the area of preventive care.

## Introduction

It has been reported that the adult population may not receive the recommended healthcare. Differences may exist between the actual and the desirable pattern of care [1]–[5], and it has been suggested that the quality of hospital care for acute and chronic condition sand for fundamental preventive services can be significantly improved [1]–[3]. Accordingly, with the aim of improving healthcare quality, the Joint Commission on Accreditation of Healthcare Organizations (JCAHO) and the Centers for Medicare & Medicaid Services (CMS) developed a uniform set of indicators that reflect the healthcare quality current evidence and practice guidelines. The quality indicators are intended to objectively measure hospital performance and to identify areas where processes of care can be improved [6]–[9]. Although adherence to practice guidelines is supposed to be associated with improved patient outcomes, persistent differences in the quality of care as well as care disparities still remain [5], [7], [10]–[13].

In Italy little is known about measurement of quality of healthcare based on a standardized set of indicators. In 2002, the National Agency for Regional Health Services (Agenzia Nazionale per i Servizi Sanitari Regionali – Age.Na.S.) was committed to identify, test and validate a set of process and outcome indicators in order to measure the quality of healthcare and community health services [14]. Indeed, a structural reform of the National Health System (NHS) is underway in Italy – the so called *devolution process* – that provides for delegation of economic and organizational authority on health to each regional government [15]. Even though equity of care and access to health services are granted to all citizens on the whole country, the ongoing health reform has yielded to a fragmentation of the NHS into 20 different Regional Health Systems. Each region has the power to legislate on the subject of health and, thus, inter-regional disparities may exist with regards to the quality of supplied healthcare.

So far, some of the Age.Na.S. indicators have been used in few regions of Italy, mainly to evaluate processes and outcomes on selected areas of health services, but the quality of healthcare has been poorly evaluated especially in the southern regions of Italy.

Thus, the purposes of the present study were to evaluate the adaptability of the JCAHO/CMS quality indicators in a geographical area of Italy and, accordingly, to obtain an estimate of adherence to selected sets of quality indicators. Moreover, these indicators can serve as a convenient and effective evaluation tool to assess disparities on receiving the optimal level of care among subgroups of population.

## Materials and Methods

### Data Collection

The medical records of all patients who were aged 18 or older admitted in one teaching-hospital and one non-teaching hospital during a one-year period, were retrospectively reviewed. Those selected are the most important public hospitals that covers the healthcare needs of the 368,000 inhabitants of the Catanzaro province (15,000 Km^2^) in the Calabria Region (2 million inhabitants) exerting a great attraction in terms of offering health services, which justifies the relevant intra-regional passive mobility.

The volume of patients treated in each hospital were almost 23,000 patients/year (occupation rate 86.2%) in the non-teaching and 4,220 (occupation rate 65.5%) in the teaching hospital; the rate of use of the medical areas was comparable with a value approximately of 67%, while in surgical settings, an higher percentage of utilization was detectable in the non-teaching hospital (100,7% vs 40%).

Data were abstracted from charts that had been selected according to the lists of ICD-9-CM principal diagnosis codes. The JCAHO/CMS measures focusing on processes of care for acute myocardial infarction (AMI) (9 indicators), heart failure (HF) (4 indicators), pneumonia (PN) (7 indicators), and surgical care improvement project (SCIP) (8 indicators) have been used (see *Appendix S1*). The standardized data collection was performed by two physicians not involved in patient care, previously trained and assessed in the ability to use the specification manual released at the time of study [16]. A detailed protocol has been used to train reviewers to abstract data from medical records in order to enhance their ability to understand key areas of the abstraction form and coding instructions. In the protocol were also included some examples that simulate the most common situations that the reviewers could find in the reality. Finally, the first 20 medical records were reviewed together by two physicians and all discrepancies were resolved through discussion, re-reading and possible intervention of a third reviewer.

Moreover, the following data were recorded for each patient: socio-demographic characteristics (gender, age, marital status, working activity, distance from home to the hospital), information on hospitalization (date, ward, source, and type of admission, date of discharge, previous admissions in the previous year), and eventual comorbidities in order to calculate the age-adjusted Charlson et al. index [17].

### Statistical Analysis

For each indicator, frequencies or medians were calculated as appropriate. Frequencies are presented as a proportion in which the number of patients who satisfy the condition of a specific indicator is divided by the total eligible population. Continuous variables are presented as the median value of the indicator for all patients who were eligible for a given measure. For the purposes of the analyses, a composite measure has been calculated for each set of quality indicators by dividing the number of achieved interventions by the number of indicators for which a patient was eligible. Thus, the numerator included the number of processes of care actually provided to a single patient, while the denominator included the number of interventions for which the patient was eligible. For continuous variable indicators, such as time from admission to antibiotics administration for pneumonia patients, the correspondent frequency-based standard has been taken into account. The resulting percentages represented the average adherence to quality indicators, and ranged from 0 to 100 depending on the number of indicators met across all measures within a disease. Calculating the risk-adjusted outcome index was beyond the purposes of this study, therefore the outcome indicator for mortality was excluded from the computation for AMI composite measure.

Backward-stepwise linear regression analyses were performed to identify selected patients’ socio-demographic characteristics independently associated with the following four outcomes of interest: adherence to AMI indicators, adherence to HF indicators, adherence to PN indicators, and adherence to SCIP indicators. The disease-specific composite measures were used as outcome variables in the models. The following independent variables were included in all models: gender (0 = male,1 = female), age (continuous in years), patient’s distance from home to hospital (continuous in kilometers), day of week of admission (0 = weekday, 1 = weekend), age-adjusted Charlson et al. co-morbidity index (0 = 0,1 = ≥1), and hospital admissions in the previous year (0 = none,1 = yes). A backward elimination procedure was applied by setting at p = 0.2 the significance level for including and at p = 0.4 for dropping variables from the models. All analyses were programmed in Stata release 11 [18].

The Ethics Committee of the “Mater Domini” Hospital of Catanzaro (Italy) approved the protocol of the study (Prot.E.C.No.2007/164). Considering the nature of the present study, which was based on reviewing medical records of discharged patients, no written consent was needed by the patients.

## Results

A total of 1772 medical records was reviewed, of which 473 (26.7%) for AMI indicators, 613 (34.6%) for HF indicators, 137 (7.7%) for PN indicators, and 549 (31%) for SCIP indicators. The selected medical records were related to patients admitted to the following wards: Cardiology, Internal Medicine, Geriatrics, Respiratory Medicine, Intensive Care Unit, Cardiac Intensive Care Unit, Cardiac Surgery, Orthopedic Surgery, General Surgery, Gynecology, Vascular Surgery. A total of 378 medical records from the teaching-hospital and 1394 from the non-teaching hospital was reviewed. Medical records selected from surgical wards were 172 e 377, respectively, for teaching and non-teaching hospital with a significantly greater adherence to SCIP indicators in the non-teaching hospital (t-test = −9.99, 547 df, p-value<0.001) (data not shown).

Table 1 shows the main characteristics of the study population. One-fourth of patients was admitted electively and was referred by the general practitioner. The remainder patients were hospitalized in emergency, of whom 20% were transferred from other hospitals. More than half were admitted in medical wards and had an age-adjusted Charlson et al. index at least of 1. One-third was hospitalized at least once in the previous year. Overall, 76 (4.3%) patients expired during hospitalization, of whom 70 (90.1%) had been admitted in urgency. Among these, 19 (27.1%) patients had a diagnosis of AMI, 34 (48.6%) were treated for HF, 9 (12.9%) were admitted for PN, and 8 (11.4%) underwent a surgical procedure. It is worth to note that all deceased patients who were treated for AMI, HF, and PN had been admitted in urgency. Mortality rates related to admission diagnosis were 4% for AMI, 5.6% for HF, 6.5% for PN, and 2.6% for SCIP.

**Table 1:** Selected characteristics of the study population.

Characteristic	No.	%
*Sex*		
Male	934	52.7
Female	838	47.3
*Age, years* *	69.4±13.9	
*Marital status*		
Married	1285	75.2
Other	423	24.8
*Patient's distance home-hospital, km* *	64.6±170.3	
*Working activity*		
Retired	1141	77.1
Other	339	22.9
*Ward of admission*		
General medicine	616	34.8
Medical specialties	318	17.9
General surgery	131	7.4
Surgical specialties	378	21.3
ICU/ED§	329	18.6
*Day of the week of admission*		
Weekday	1475	83.2
Weekend	297	16.8
*Admission source*		
Emergency Department	960	54.2
General practitioner	443	25
Other hospital	259	14.6
Other	110	6.2
*Type of admission*		
Emergency	1319	74.4
Elective	453	25.6
*Length of stay, days* *	10.7±7.6	
*Age-adjusted Charlson comorbidity index*		
0	782	44.1
≥1	990	55.9
*Previous admissions in the previous year*		
0	1203	67.9
≥1	569	32.1

* Values are expressed as mean ± SD.

§ ICU/ED = Intensive Care Units/Emergency Departments.

### Adherence to AMI Indicators

Appropriate prescription of aspirin at arrival and at discharge, and of angiotensin-converting enzyme inhibitor (ACEI) or angiotensin receptor blocker (ARB) were provided to more than 90% of the eligible patients. Lower compliance ranging from 25% for primary percutaneous cardiac intervention (PCI) received within 90 minutes to 65.1% for appropriate beta-blocker prescription. On average, 70% (±25.2 SD) of eligible patients received the recommended processes of care (*Table 2*). Multiple linear regression analysis showed that age had a significant negative relationship with adherence to AMI (β = −0.19, p-value = 0.04) *(data not shown).*

**Table 2:** Frequency distribution of quality measures evaluated.

Measure	No. of eligible patients	Median time indicator	% of eligible patients who met the indicator
***Acute Myocardial Infarction (473)°***			
Aspirin at arrival	222		92.8
Aspirin prescribed at discharge	360		97.2
ACEI or ARB for LVSD	59		94.9
Adult smoking cessation advice/counseling§	224		0
Beta-blocker prescribed at discharge	340		75.6
Beta-blocker at arrival	195		65.1
Median time to fibrinolysis, minutes	81	28	
Fibrinolytic therapy received within 30 minutes of hospital arrival	81		53.1
Median time to primary PCI, minutes	4	205	
Primary PCI received within 90 minutes of hospital arrival	4		25
Inpatient mortality	473		4
*AMI composite measure (%)* a b		*70±25.2*	
***Heart Failure (613)°***			
Discharge instructions	579		0
Evaluation of LVS function	533		77.5
ACEI or ARB for LVSD	201		93.5
Adult smoking cessation advice/counseling§§	391		0.5
*HF composite measure (%)* b		*32.4±22.8*	
***Pneumonia (137)°***			
Oxygenation assessment	134		63.4
Pneumococcal vaccination	67		0
Blood cultures performed within 24 hours prior to or 24 hours after hospital arrival forpatients who were transferred or admitted to the ICU within 24 hours of hospital arrival	1		100
Blood cultures performed in the emergency department prior to initial antibiotic receivedin hospital	0		-
Adult smoking cessation advice/counseling§§§	85		0
Antibiotic timing, minutes	16	182	
Initial antibiotic received within 4 hours of hospital arrival	16		68.8
Initial antibiotic received within 6 hours of hospital arrival	16		68.8
Initial antibiotic selection for CAP in immunocompetent patient	127		66.1
Initial antibiotic selection for CAP in immunocompetent – ICU patient	0		-
Initial antibiotic selection for CAP in immunocompetent – non ICU patient	127		66.1
Influenza vaccination	24		0
*PN composite measure (%)* b		*46.4±29.5*	
***Surgical Care Improvement Project (549)°***			
Prophylactic antibiotic received within one hour prior to surgical incision - overall rate	547		9.1
Prophylactic antibiotic selection for surgical patients - overall rate	542		11.8
Prophylactic antibiotics discontinued within 24 hours after surgery end time - overall rate	542		2.2
Cardiac surgery patients with controlled 6 a.m. postoperative blood glucose	78		60.3
Surgery patients with appropriate hair removal	450		94.9
Colorectal surgery patients with immediate postoperative normothermia	105		87.6
Surgery patients on beta-blocker therapy prior to arrival who received a beta-blockerduring the perioperative period	74		55.4
Surgery patients with recommended venous thromboembolism prophylaxis ordered	374		99.5
Surgery patients who received appropriate venous thromboembolism prophylaxis within24 hours prior to surgery to 24 hours after surgery	374		99.5
*SCIP composite measure (%)* b		*46±16.3*	

AMI = acute myocardial infarction; HF = heart failure; PN = pneumonia; SCIP = surgical care improvement project; ACEI = angiotensin-converting enzyme inhibitors; ARB = angiotensin receptor blockers; LSVD = left systolic ventricular dysfunction; PCI = percutaneous cardiac intervention; ICU = intensive care unit; ED = emergency department; CAP = community-acquired pneumonia.

*°*In brackets is reported the overall number of patients for each set of measures.

§ Not documented in 122 (54.5%) medical records.

§§ Not documented in 367 (93.9%) medical records.

§§§ Not documented in 72 (84.7%) medical records.

a AMI inpatient mortality not included for the calculation of the composite measure.

b Values are expressed as mean ± SD.

### Adherence to HF Indicators

ACEI or ARB were prescribed at discharge in 93.5% of the eligible patients, and left ventricular function assessment was documented in 77.5%. Complete discharge instructions were provided to none of the eligible patients. The mean composite score was 32.4% (±22.8 SD) (*Table 2*). Multivariate analysis showed that age was the only predictor negatively associated with adherence to HF indicators (β = −0.60, p-value<0.0001) (Table 3).

**Table 3:** Results of linear regression analyses.

Model 1: adherence to acute myocardial infarction indicators F (1, 434) = 4.25; p-value = 0.04; R^2^ = 0.0097; Adjusted R^2^ = 0.01				
*Variable*	*COEFF*	*SE*	*t*	*P*
Age	−0.19	0.09	−2.06	0.040
Constant	82.92	6.36	13.03	<0.0001
**Model 2: adherence to heart failure indicators F (4, 574) = 12.51; p-value<0.0001; R^2^ = 0.08; Adjusted R^2^ = 0.07**				
*Variable*	*COEFF*	*SE*	*t*	*P*
Age	−0.60	0.10	−6.58	<0.0001
Gender	3.56	1.86	1.91	0.056
Distance home-hospital	0.02	0.01	1.11	0.267
Day of the week of admission	−2.03	2.23	−0.91	0.362
Constant	76.49	7.00	10.93	<0.0001
**Model 3: adherence to surgical care improvement project indicators F(3, 545) = 10.75; p-value<0.0001; R^2^ = 0.06; Adjusted R^2^ = 0.05**				
*Variable*	*COEFF*	*SE*	*t*	*P*
Age-adjusted Charlson comorbidity index	−4.64	1.43	−3.25	0.001
Age	−0.11	0.05	−2.07	0.039
Patient's distance home-hospital	−0.01	0.01	−1.74	0.082
Constant	0.57	0.01	58.61	<0.0001

### Adherence to PN Indicators

None of the eligible patients was provided with pneumococcal or influenza vaccination, or smoking cessation advice either. For all other indicators, adherence never reached 70%. The mean percentage of composite score was 46.4% (±29.5 SD) (*Table 2*). The sample size for the adherence to PN indicators was quite small, and no significant relationship was found on multivariate analysis (data not shown).

### Adherence to SCIP Indicators

High compliance was revealed only for four indicators: the two related to thromboembolism prophylaxis (99.5%), the appropriate hair removal in surgery patients (94.9%), and the assessment of post-operative normothermia in colorectal surgery patients (87.6%). An extreme variability of compliance was ascertained for all other indicators, ranging from 2.2% for timely stopping of prophylactic antibiotics to 60.3% for postoperative blood glucose testing. The mean percentage of the composite score was 46% (±16.3 SD) (*Table 2*). Results from multivariate analysis showed that both age-adjusted Charlson et al. index (β = −4.64, p-value = 0.001) and distance from patient's home to hospital (β = −0.11, p-value = 0.039) had a negative relationship with adherence to SCIP indicators (*Table 3*).

## Discussion

The present study is intended to be an analytical first step in measuring the quality of hospital care in an area of Italy by using a set of indicators that reflect the adherence to current evidence-based processes of care. The application of JCAHO/CMS quality indicators has provided valuable insight into their feasibility, ease of use, and availability of required data. This experience indicates that these quality indicators can be implemented in this context, and they showed to be easy to use.

The results of this study show that quality of hospital care is extremely variable according to indicators and to conditions and is often inadequate. Composite scores indicate that patients may not receive the recommended care in many cases and that there is wide room for improvement. Actually, one could expect a wide-ranging adherence rates at the baseline measurement, since this tendency has already been reported in earlier works on the topic. Indeed, in a study reporting time-series data over eight quarters from 2002 through 2004 in the U.S. hospitals, a wide variation in adherence to quality indicators was reported at the baseline measurement, whereas a significant compliance improvement for 15 of the 18 indicators was recorded over time [19].

This baseline measurement, according to its observational nature, allowed us to perform a real-world assessment of patterns of care, before any quality improvement initiative had been undertaken, and showed a very challenging scenario that deserves careful interpretation. Indeed, only few measures (use of aspirin and ACEI or ARB for AMI, ACEI or ARB for HF, appropriate thromboembolism prophylaxis and appropriate hair removal for surgical patients) almost approached optimal adherence, whereas, at the other extreme, rates regarding adherence to smoking-cessation counseling in AMI and HF patients, discharge instructions in HF patients, and influenza and pneumococcal vaccination in pneumonia patients were noticeably intangible. For all other measures a wide variation in uptake was registered, regardless of the condition taken into account. This variability has already been reported by Jha et al. [20] in American hospitals, where for five indicators related to AMI half of the hospitals scored over 90%, whereas the level of performance for the other measures was much lower and variable.

A number of studies have identified potential barriers and factors for the adoption of best-practice guidelines. Reasons underlying this wide variation in adherence to quality indicators can be different and can be related to individual, organizational, and environmental factors [21]. It has been suggested that variation in compliance to recommended processes of care may reflect differences in training, guideline familiarity, and implementation of tools and systems to ensure that recommended care is provided and documented [22], [23]. Indeed, another factor affecting the adherence to quality indicators may be the impaired perception about connection of evidence-based processes of care to improved outcomes [6], [8], [24], [25]. Two surveys conducted by some of us among Italian physicians documented that, despite a general agreement towards the need to integrate clinical practice and the best available evidence, they not frequently used results of economic evaluations, RCTs and meta-analyses to make decisions in the clinical practice [26]. These results are quite consistent with those of another investigation regarding Italian general practitioners' perceptions of Evidence Based Medicine and its influence on headache patient management [27]. However, it is difficult to translate evidence into clinical continuing educational programs and, therefore, raising awareness of how to use tools to critically appraise and apply the evidence to their patients are strongly needed [28]. Furthermore, a number of studies showed that hospitals’ characteristics such as type, size, availability of given technologies and services, and geographic factors can play a role in the uptake of evidence-based processes of care [6], [21], [29]–[32], along with the capability in fitting and customization of existing guidelines to local contexts [33]. In these settings many of these barriers may have had a role, but it is possible to tentatively try to suggest reasons for lack of adherence to some of the measured indicators. The pattern of performance observed seems to confirm previous research that showed how quality performance may vary more by functional roles in the hospital, such as treatment and diagnosis vs counseling and prevention, than by a particular disease being treated [34]. This is in agreement with the findings of this study, where preventive indicators were those that can receive the largest improvements. This is of concern, since some of these indicators relates to effective practices, such as, for instance, patient education for the treatment of HF [22], [35]; moreover, from a hospital management perspective, interest in performance is related to both clinical (i.e. prescriptions and/or treatment procedures) and preventive (i.e. discharge HF education, vaccination practices and/or counseling on known risk factors) care.

It should also be noted that, at least partly, recommended processes of care were actually supplied but were not detailed in medical records. Thus, the low adherence rates to some evidence-based measures may underestimate the real uptake, mainly, for appropriate timing and selection of prophylactic antibiotics in surgical patients. Moreover, adherence to blood cultures (BCs) indicators was also inconsistent, which may reflect physicians’ awareness that BCs may have a limited utility in community-acquired pneumonia (CAP) patients. A systematic review of cohort studies showed that true-positive values of BCs obtained at hospital admission from patient admitted for CAP ranged from 0% to 14% of cases [36].

The present study was designed to provide information on process indicators and not on patient outcomes. Although all of the performance indicators measured were derived from the JCAHO and the CMS set of indicators and reflect the healthcare quality current evidence and practice guidelines, variable associations between performance measures and outcomes have been reported by several studies. Indeed, Wang et al. found that hospitals with better performance on both AMI and HF measures had lower risk-adjusted mortality compared with hospitals adherent to neither or either alone [37], whereas Ingraham et al. found only partial association between adherence to SCIP indicators and risk-adjusted outcomes related to morbidity and mortality following surgery [38].

In-hospital mortality rates can be regarded as a measure of association of hospital adherence to guidelines and patient outcomes. In this study, the overall mortality rate was 4%, whereas with respect to the principal diagnosis of admission, mortality rates ranged from 2.6% to 6.5%. These rates were steadily lower than those published in the Age.Na.S’s study [14], since condition-specific mortality rates were 7.5% for AMI, 7.1% for HF, and 8.6 for PN. It is plausible that these differences in mortality may be due to the fact that the Age.Na.S study [14] refers to condition-specific mortality, while results of the present study concerned the in-hospital mortality of patients admitted with one of the four principal diagnosis selected. As regard the association between adherence to indicators and in-hospital mortality the only for which it was plausible to make this assessment were AMI and HF indicators. Indeed, for PN and SCIP the procedures identified by the indicators could, at most, affect the long-term mortality and could hardly be related to in-hospital mortality. Therefore, it was possible to model only the mortality for AMI as the outcome variable and a significant association has been found with a lowest adherence to AMI indicators (OR = 0.97; 95% CI = 0.95–0.99; p = 0.032) (data not shown). Although this was not an aim of this study, it may be suggested that effectiveness of process indicators should be more thoroughly investigated in the real world.

Most of the previous studies were conducted on large numbers of hospitals and therefore were based on aggregated data. Instead, the results in this study were derived from a smaller number of patients, but detailed information was gathered from each of them. This was a strength of this study and allowed us to indicate subjective characteristics that could predict adherence to performance indicators. Indeed, the results from the multivariate analyses showed that age significantly predicted the adherence to quality indicators for AMI, HF, and SCIP, since older patients were less likely to receive the recommended processes of care. The findings are consistent with those reported by some authors who found out that patients aged ≥75 were independently associated with a lower level of care and worse outcomes [7], [39]. None of the other socio-demographic characteristics appear to influence the behavior of health professionals in the application of indicators. Further research is needed, involving larger datasets, that will identify eventual other subject or hospital characteristics that are related to the appropriateness of the process of care.

Some potential limitations of the present study need to be acknowledged. First, comparisons across countries should be made cautiously, since it has to do with the appraisal of different healthcare systems. Second, a main shortcoming may arise from the lack of follow up and it was not possible to appraise any relationship with indicators and outcomes over time. However, it was not an objective of the present study that was, instead, to detect an estimate of adherence to selected process of care indicators as a measure of quality of care provided in-hospital setting. Finally, it should be noted that the results depend not only on the quality of care provided, but also on patient characteristics that may be outside the direct control of a hospital [9]. Therefore, it is not possible to report any change over time. Indeed, data abstraction was sharply critical in many cases, since it was not possible to retrieve the necessary data from medical records or the medical files were not available at all. Thus, it is arguable that availability and quality of data may have contributed to lower estimates of the adherence rates. Third, despite the importance of the patients educational level in the adherence to the treatments they undergo [40], this information was not present in the medical record and, therefore, the study does not provide guidance in this regard. Finally, although the reviewers collected the data not blinded to the outcome of interest, the use of explicit and objective indicators that relies entirely on the presence or absence of specific information entails that there is no influence of reviewers on the quality of abstracted data.

### Conclusions

The wide variation and in some instances the very low adherence to quality indicators suggests that there is still substantial work that lies ahead on the way to improve hospital performance. Efforts should focus more on domains of healthcare than on specific conditions, and particularly on improvement in preventive care. Moreover, resources should be devoted to expand comprehensiveness and quality of data in medical records and to identify specific subgroup of the population that need a special attention in delivering care.

## Supporting Information

Appendix S1 Quality indicators for acute myocardial infarction, heart failure, pneumonia and surgical care improvement (Adapted from Williams SC et al., NEJM 2005;353∶255–264). ACE = angiotensin-converting enzyme; ARB = angiotensin receptor blocker; PCI = percutaneous coronary intervention; ICU = intensive care unit; CABG = coronary artery bypass grafting
(DOC)

## References

[1] Jencks SF, Huff ED, Cuerdon T (2003) Change in the quality of care delivered to Medicare beneficiaries, 1998–1999 to 2000–2001. JAMA 289: 305–312. 12525231 10.1001/jama.289.3.305

[2] McGlynn EA, Asch SM, Adams J, Keesey J, Hicks J, et al (2003) The quality of health care delivered to adults in the United States. N Engl J Med 348: 2635–2645. 12826639 10.1056/NEJMsa022615

[3] Kerr EA, McGlynn EA, Adams J, Keesey J, Asch SM (2004) Profiling the quality of care in twelve communities: results from the CQI study. Health Aff (Millwood) 23: 247–256. 15160823 10.1377/hlthaff.23.3.247

[4] Schuster MA, McGlynn EA, Brook RH (2005) How good is the quality of health care in the United States? 1998. Milbank Q 83: 843–895. 16279970 10.1111/j.1468-0009.2005.00403.x PMC2690270

[5] Asch SM, Kerr EA, Keesey J, Adams JL, Setodji CM, et al (2006) Who is at greatest risk for receiving poor-quality health care? N Engl J Med 354: 1147–1156. 16540615 10.1056/NEJMsa044464

[6] Peterson ED, Roe MT, Mulgund J, DeLong ER, Lytle BL, et al (2006) Association between hospital process performance and outcomes among patients with acute coronary syndromes. JAMA 295: 1912–1920. 16639050 10.1001/jama.295.16.1912

[7] Peterson ED, Shah BR, Parsons L, Pollack CV Jr, French WJ, et al (2008) Trends in quality of care for patients with acute myocardial infarction in the National Registry of Myocardial Infarction from 1990 to 2006. Am Heart J 156: 1045–1055. 19032998 10.1016/j.ahj.2008.07.028

[8] Maeda JL (2010) Evidence-based heart failure performance measures and clinical outcomes: a systematic review. J Card Fail 16: 411–418. 20447578 10.1016/j.cardfail.2010.01.005

[9] Tu JV, Khalid L, Donovan LR, Ko DT (2008) Indicators of quality of care for patients with acute myocardial infarction. CMAJ 179: 909–915. 18936456 10.1503/cmaj.080749 PMC2565729

[10] Hasnain-Wynia R, Baker DW, Nerenz D, Feinglass J, Beal AC, et al (2007) Disparities in health care are driven by where minority patients seek care: examination of the hospital quality alliance measures. Arch Intern Med 167: 1233–1239. 17592095 10.1001/archinte.167.12.1233

[11] Bernheim SM, Spertus JA, Reid KJ, Bradley EH, Desai RA, et al (2007) Socioeconomic disparities in outcomes after acute myocardial infarction. Am Heart J 153: 313–319. 17239695 10.1016/j.ahj.2006.10.037

[12] Kosiak B, Sangl J, Correa-de-Araujo R (2006) Quality of health care for older women: what do we know? Womens Health Issues 16: 89–99. 16638525 10.1016/j.whi.2005.01.003

[13] Correa-de-Araujo R, Stevens B, Moy E, Nilasena D, Chesley F, et al (2006) Gender differences across racial and ethnic groups in the quality of care for acute myocardial infarction and heart failure associated with comorbidities. Womens Health Issues 16: 44–55. 16638521 10.1016/j.whi.2005.04.003

[14] Agenzia nazionale per i servizi sanitari regionali, AgeNaS (2005) Identificazione, sperimentazione e validazione di alcuni indicatori di processo ed esito della qualità delle attività sanitarie. Rapporto conclusivo. Supplemento al n.15 di Monitor. Elementi di analisi e ossevazione del sistema salute. Available: http://www.agenas.it/agenas_pdf/SupplMon15_Indicatori.zip. Accessed 2012 Aug 25.

[15] Osservatorio nazionale sulla salute nelle regioni italiane. Rapporto Osservasalute 2009. Available: http://www.osservasalute.it/index.php/rapporto/argomenti/2009/8. Accessed 2012 Aug 25.

[16] QualityNet.Org (2010) Specifications Manual for National Hospital Inpatient Quality Measures. Version 3.1.a, 2010. Available: http://www.qualitynet.org/dcs/ContentServer?c=Page&pagename=QnetPublic%2FPage%2FQnetTier4&cid=1228749003528. Accessed 2010 Jun 28.

[17] Charlson ME, Pompei P, Ales KL, MacKenzie CR (1987) A new method of classifying prognostic comorbidity in longitudinal studies: development and validation. J Chronic Dis 40: 373–383. 3558716 10.1016/0021-9681(87)90171-8

[18] StataCorp (2009) Stata: Release 11. Statistical Software. College Station; TX: StataCorp LP.

[19] Williams SC, Schmaltz SP, Morton DJ, Koss RG, Loeb JM (2005) Quality of care in U.S. hospitals as reflected by standardized measures, 2002–2004. N Engl J Med 353: 255–264. 16034011 10.1056/NEJMsa043778

[20] Jha AK, Li Z, Orav EJ, Epstein AM (2005) Care in U.S. hospitals–the Hospital Quality Alliance program. N Engl J Med 353: 265–274. 16034012 10.1056/NEJMsa051249

[21] Ploeg J, Davies B, Edwards N, Gifford W, Miller PE (2007) Factors influencing best-practice guideline implementation: lessons learned from administrators, nursing staff, and project leaders. Worldviews Evid Based Nurs 4: 210–219. 18076464 10.1111/j.1741-6787.2007.00106.x

[22] Fonarow GC, Yancy CW, Heywood JT (2005) Adherence to heart failure quality-of-care indicators in US hospitals: analysis of the ADHERE Registry. Arch Intern Med 165: 1469–1477. 16009861 10.1001/archinte.165.13.1469

[23] Cabana MD, Rand CS, Powe NR, Wu AW, Wilson MH, et al (1999) Why don't physicians follow clinical practice guidelines? A framework for improvement. JAMA 282: 1458–1465. 10535437 10.1001/jama.282.15.1458

[24] Bradley EH, Herrin J, Elbel B, McNamara RL, Magid DJ, et al (2006) Hospital quality for acute myocardial infarction: correlation among process measures and relationship with short-term mortality. JAMA 296: 72–78. 16820549 10.1001/jama.296.1.72

[25] Huynh LT, Chew DP, Sladek RM, Phillips PA, Brieger DB, et al (2009) Unperceived treatment gaps in acute coronary syndromes. Int J Clin Pract 63: 1456–1464. 19769702 10.1111/j.1742-1241.2009.02182.x

[26] De Vito C, Nobile CG, Furnari G, Pavia M, De Giusti M, et al (2009) The role of education in improving physicians’ professional use of economic evaluations of health interventions. Some evidence from a cross-sectional survey in Italy. Eval Health Prof 32: 249–263. 19679635 10.1177/0163278709338557

[27] Bianco A, Parente MM, De Caro E, Iannacchero R, Cannistrà U, et al (2005) Evidence-based medicine and headache patient management by general practitioners in Italy. Cephalalgia 25: 767–775. 16162253 10.1111/j.1468-2982.2005.00972.x

[28] De Vito C, Nobile CG, Furnari G, Pavia M, De Giusti M, et al (2009) Physicians’ knowledge, attitudes and professional use of RCTs and meta-analyses: A cross-sectional survey. Eur J Public Health 19: 297–302. 19129347 10.1093/eurpub/ckn134

[29] Joynt KE, Harris Y, Orav EJ, Jha AK (2011) Quality of care and patient outcomes in critical access rural hospitals. JAMA 306: 45–52. 21730240 10.1001/jama.2011.902 PMC3337777

[30] Nunez-Smith M, Bradley EH, Herrin J, Santana C, Curry LA, et al (2011) Quality of care in the US territories. Arch Intern Med 171: 1528–1540. 21709184 10.1001/archinternmed.2011.284 PMC3251926

[31] Popescu I, Nallamothu BK, Vaughan-Sarrazin MS, Cram P (2008) Do specialty cardiac hospitals have greater adherence to acute myocardial infarction and heart failure process measures? An empirical assessment using Medicare quality measures: quality of care in cardiac specialty hospitals. Am Heart J 156: 155–160. 18585511 10.1016/j.ahj.2008.02.018

[32] Fox KA, Goodman SG, Anderson FA Jr, Granger CB, Moscucci M, et al (2003) From guidelines to clinical practice: the impact of hospital and geographical characteristics on temporal trends in the management of acute coronary syndromes. The Global Registry of Acute Coronary Events (GRACE). Eur Heart J 24: 1414–1424. 12909070 10.1016/s0195-668x(03)00315-4

[33] Harrison MB, Legare F, Graham ID, Fervers B (2010) Adapting clinical practice guidelines to local context and assessing barriers to their use. CMAJ 182: E78–84. 19969563 10.1503/cmaj.081232 PMC2817341

[34] Landon BE, Normand SL, Lessler A, O'Malley AJ, Schmaltz S, et al (2006) Quality of care for the treatment of acute medical conditions in US hospitals. Arch Intern Med 166: 2511–2517. 17159018 10.1001/archinte.166.22.2511

[35] Hunt SA, Baker DW, Chin MH, Cinquegrani MP, Feldman AM, et al (2001) ACC/AHA guidelines for the evaluation and management of chronic heart failure in the adult: executive summary. A report of the American College of Cardiology/American Heart Association Task Force on Practice Guidelines (Committee to revise the 1995 Guidelines for the Evaluation and Management of Heart Failure). J Am Coll Cardiol 38: 2101–2113. 11738322 10.1016/s0735-1097(01)01683-7

[36] Afshar N, Tabas J, Afshar K, Silbergleit R (2009) Blood cultures for community-acquired pneumonia: are they worthy of two quality measures? A systematic review. J Hosp Med 4: 112–123. 19219920 10.1002/jhm.382

[37] Wang TY, Dai D, Hernandez AF, Bhatt DL, Heidenreich PA, et al (2011) The importance of consistent, high-quality acute myocardial infarction and heart failure care results from the American Heart Association's Get with the Guidelines Program. J Am Coll Cardiol 58: 637–644. 21798428 10.1016/j.jacc.2011.05.012

[38] Ingraham AM, Cohen ME, Bilimoria KY, Dimick JB, Richards KE, et al (2010) Association of surgical care improvement project infection-related process measure compliance with risk-adjusted outcomes: implications for quality measurement. J Am Coll Surg 211: 705–714. 21109157 10.1016/j.jamcollsurg.2010.09.006

[39] Fonarow GC, Abraham WT, Albert NM, Stough WG, Gheorghiade M, et al (2009) Age- and gender-related differences in quality of care and outcomes of patients hospitalized with heart failure (from OPTIMIZE-HF). Am J Cardiol 104: 107–115. 19576329 10.1016/j.amjcard.2009.02.057

[40] Gale N, Marshall T, Bramley G (2012) Starting and staying on preventive medication for cardiovascular disease. Curr Opin Cardiol 27: 533–41. 22820103 10.1097/HCO.0b013e328356dae5

