# Supplementary material for: Appraising Hospital Performance by Using the JCHAO/CMS Quality Measures in Southern Italy
Source: PLoS One. 2012 Nov 7;7(11):e48923. doi: 10.1371/journal.pone.0048923 (PMC3492134; doi:10.1371/journal.pone.0048923)
Supplement: Appendix S1 — Quality indicators for acute myocardial infarction, heart failure, pneumonia and surgical care improvement (Adapted from Williams SC et al., NEJM 2005;353∶255–264). ACE = angiotensin-converting enzyme; ARB = angiotensin receptor blocker; PCI = percutaneous coronary intervention; ICU = intensive care unit; CABG = coronary artery bypass grafting (DOC) [file pone.0048923.s001.doc]

**Appendix S1** **Quality indicators for acute myocardial infarction, heart failure, pneumonia and surgical care improvement.**

(Adapted from Williams SC et al., NEJM 2005;353:255-264)

| **Measure** | **Patients included** | **Patients excluded** |
| --- | --- | --- |
| **Acute myocardial infarction** |  |  |
| Aspirin within 24 hours before or after admission | Patients 18 years of age or older with a principal ICD-9-CM discharge diagnosis of acute myocardial infarction. | Patients who were transferred to another acute care hospital on day of arrival or transferred from another hospital, including another emergency department; patients who were discharged, died, or left against medical advice on day of arrival; patients with one or more contraindications to aspirin; patients who were receiving comfort measures only. |
| Aspirin prescribed at discharge | Patients 18 years of age or older with a principal ICD-9-CM discharge diagnosis of acute myocardial infarction. | Patients who were transferred to another acute care hospital, died, left against medical advice, or were discharged to a hospice; patients with one or more contraindications to aspirin; patients who were receiving comfort measures only. |
| ACE inhibitors or ARB prescribed at discharge for patients with left ventricular systolic dysfunction | Patients 18 years of age or older with a principal ICD-9-CM discharge diagnosis of acute myocardial infarction. | Patients who were transferred to another acute care hospital, died, left against medical advice, or were discharged to a hospice; patients with one or more contraindications to ACE inhibitors; patients who were receiving comfort measures only. |
| Adult smoking-cessation counseling or advice | Patients 18 years of age or older with a principal ICD-9-CM discharge diagnosis of acute myocardial infarction. | Patients who were transferred to another acute care hospital, died, left against medical advice, or were discharged to a hospice; patients who were receiving comfort measures only. |
| Beta-blocker prescribed at discharge | Patients 18 years of age or older with a principal ICD-9-CM discharge diagnosis of acute myocardial infarction. | Patients who were transferred to another acute care hospital, died, left against medical advice, or were discharged to a hospice; patients with one or more contraindications to beta-blockers; patients who were receiving comfort measures only. |
| Beta-blocker within 24 hours after admission | Patients 18 years of age or older with a principal ICD-9-CM discharge diagnosis of acute myocardial infarction. | Patients who were transferred to another acute care hospital on day of arrival or transferred from another hospital, including another emergency department; patients who were discharged, died, or left against medical advice on day of arrival; patients with one or more contraindications to beta-blockers; patients who were receiving comfort measures only. |
| Median time in minutes from arrival to thrombolysis | Patients 18 years of age or older with a principal ICD-9-CM discharge diagnosis of acute myocardial infarction. Patients with ST-segment elevation or left bundle-branch block on the electrocardiogram obtained closest to hospital arrival who received thrombolytic therapy within the first 6 hours after arrival. | Patients who were transferred to another acute care hospital on day of arrival or transferred from another hospital, including another emergency department; patients who received thrombolytic therapy more than 6 hours after arrival; patients who were receiving comfort measures only. |
| Fibrinolytic therapy received within 30 minutes of hospital arrival | Patients 18 years of age or older with a principal ICD-9-CM discharge diagnosis of acute myocardial infarction. Patients with ST-segment elevation or left bundle-branch block on the electrocardiogram obtained closest to hospital arrival who received thrombolytic therapy within the first 6 hours after arrival. | Patients who were transferred to another acute care hospital on day of arrival or transferred from another hospital, including another emergency department; patients who received thrombolytic therapy more than 6 hours after arrival; patients who were receiving comfort measures only. |
| Median time in minutes from arrival to PCI | Patients 18 years of age or older with a principal ICD-9-CM discharge diagnosis of acute myocardial infarction. Patients with ST-segment elevation or left bundle-branch block on the electrocardiogram obtained closest to hospital arrival; patients with a valid ICD-9-CM procedure code for PCI; patients who underwent PCI within the first 24 hours after arrival. | Patients who were transferred to another acute care hospital on day of arrival or transferred from another hospital, including another emergency department; patients who underwent PCI more than 24 hours after hospital arrival; patients given thrombolytic agents; patients who were receiving comfort measures only. |
| Primary PCI received within 90 minutes of hospital arrival | Patients 18 years of age or older with a principal ICD-9-CM discharge diagnosis of acute myocardial infarction. Patients with ST-segment elevation or left bundle-branch block on the electrocardiogram obtained closest to hospital arrival; patients with a valid ICD-9-CM procedure code for PCI; patients who underwent PCI within the first 24 hours after arrival. | Patients who were transferred to another acute care hospital on day of arrival or transferred from another hospital, including another emergency department; patients who received thrombolytic therapy more than 6 hours after arrival; patients who were receiving comfort measures only. |
| Inpatient death | Patients 18 years of age or older with a principal ICD-9-CM discharge diagnosis of acute myocardial infarction. | Patients who were transferred from another acute-care hospital. |
| **Heart failure** |  |  |
| Discharge instructions regarding medications, diet, weight, worsening of symptoms, follow-up, and activity | Patients 18 years of age or older with a principal ICD-9-CM discharge diagnosis of heart failure. | Patients discharged or transferred anywhere except home, home care, or home intravenous therapy; patients who were receiving comfort measures only. |
| Evaluation of left ventricular function | Patients 18 years of age or older with a principal ICD-9-CM discharge diagnosis of heart failure. | Patients who were transferred to another acute care hospital, died, left against medical advice, or were discharged to a hospice; patients with documented reasons for the absence of an assessment of left ventricular function; patients who were receiving comfort measures only. |
| ACE inhibitors or ARB prescribed at discharge for patients with left ventricular systolic dysfunction | Patients 18 years of age or older with a principal ICD-9-CM discharge diagnosis of heart failure. | Patients who were transferred to another acute care hospital, died, left against medical advice, or were discharged to a hospice; patients with one or more contraindications to ACE inhibitors; patients who were receiving comfort measures only. |
| Smoking-cessation counseling or advice | Patients 18 years of age or older with a principal ICD-9-CM discharge diagnosis of heart failure. | Patients who were transferred to another acute care hospital, died, left against medical advice, or were discharged to a hospice; patients who were receiving comfort measures only. |
| **Pneumonia** |  |  |
| Oxygenation assessment within 24 hours prior to or after arrival | Patients 18 years of age or older with a principal ICD-9-CM discharge diagnosis of pneumonia (or a principal discharge diagnosis of septicemia or respiratory failure with a secondary diagnosis code of pneumonia). | Patients less than 18 years of age; patients who were transferred from another acute care or critical care access hospital; patients who had no chest x-ray or CT scan indicating abnormal findings within 24 hours prior to hospital arrival or during this hospitalization; patients who were receiving comfort measures only. |
| Pneumococcal screening, vaccination, or both by discharge | Patients 18 years of age or older with a principal ICD-9-CM discharge diagnosis of pneumonia (or a principal discharge diagnosis of septicemia or respiratory failure with a secondary diagnosis code of pneumonia). | Patients less than 65 years of age; patients who were transferred from another acute care including emergency department; patients who were transferred to another acute care hospital, died, left against medical advice, or were discharged to a hospice; patients who had no chest x-ray or CT scan indicating abnormal findings within 24 hours prior to hospital arrival or during this hospitalization; patients who were receiving comfort measures only. |
| Blood cultures performed within 24 hours prior to or after arrival for patients who were transferred or admitted to ICU | Patients 18 years of age or older with a principal ICD-9-CM discharge diagnosis of pneumonia (or a principal discharge diagnosis of septicemia or respiratory failure with a secondary diagnosis code of pneumonia). | Patients less than 18 years of age; patients who were transferred from another acute care or critical care access hospital; patients who had no chest x-ray or CT scan indicating abnormal findings within 24 hours prior to hospital arrival or during this hospitalization; patients who were receiving comfort measures only; patients for whom no blood cultures were obtained. |
| Blood cultures collected in Emergency Department before initiation of antibiotic therapy in hospital | Patients 18 years of age or older with a principal ICD-9-CM discharge diagnosis of pneumonia (or a principal discharge diagnosis of septicemia or respiratory failure with a secondary diagnosis code of pneumonia). | Patients less than 18 years of age; patients who were transferred from another acute care or critical care access hospital; patients who had no chest x-ray or CT scan indicating abnormal findings within 24 hours prior to hospital arrival or during this hospitalization; patients who were receiving comfort measures only; patients for whom no blood cultures were obtained. |
| Adult smoking-cessation counseling or advice | Patients 18 years of age or older with a principal ICD-9-CM discharge diagnosis of pneumonia (or a principal discharge diagnosis of septicemia or respiratory failure with a secondary diagnosis code of pneumonia). | Patients less than 18 years of age; patients who were transferred to another acute care hospital, died, left against medical advice, or were discharged to a hospice; patients who had no chest x-ray or CT scan indicating abnormal findings within 24 hours prior to hospital arrival or during this hospitalization; patients who were receiving comfort measures only. |
| Median time from arrival to initial antibiotic administration | Patients 18 years of age or older with a principal ICD-9-CM discharge diagnosis of pneumonia (or a principal discharge diagnosis of septicemia or respiratory failure with a secondary diagnosis code of pneumonia). | Patients less than 18 years of age; patients who were transferred from another acute care or critical care access hospital; patients who had no chest x-ray or CT scan indicating abnormal findings within 24 hours prior to hospital arrival or during this hospitalization; patients who were receiving comfort measures only; patients who did not receive antibiotics during hospitalization or whose initial antibiotic was administered more than 36 hours after arrival. |
| Initial antibiotic selection for community-acquired pneumonia (CAP) in immunocompetent patients | Patients 18 years of age or older with a principal ICD-9-CM discharge diagnosis of pneumonia (or a principal discharge diagnosis of septicemia or respiratory failure with a secondary diagnosis code of pneumonia). | Patients less than 18 years of age; patients who were transferred from another acute care or critical care access hospital; patients who had no chest x-ray or CT scan indicating abnormal findings within 24 hours prior to hospital arrival or during this hospitalization; patients who were receiving comfort measures only; patients who did not receive any antibiotics within 24 hours after arrival; patients who were transferred to another acute care hospital, died, left against medical advice, or were discharged to a hospice; patients who are immunocompromised. |
| Influenza vaccination | Patients 18 years of age or older with a principal ICD-9-CM discharge diagnosis of pneumonia (or a principal discharge diagnosis of septicemia or respiratory failure with a secondary diagnosis code of pneumonia). | Patients less than 50 years of age; patients who had no chest x-ray or CT scan indicating abnormal findings within 24 hours prior to hospital arrival or during this hospitalization; patients who were receiving comfort measures only; patients with a secondary diagnosis of 487.0; patients who were transferred to another acute care hospital, died, left against medical advice, or were discharged to a hospice. |
| **Surgical care improvement project** |  |  |
| Prophylactic antibiotic received within one hour prior to surgical incision - overall rate | Patients 18 years of age or older with a principal ICD-9-CM discharge diagnosis of CABG, other cardiac surgery, hip or knee arthroplasty, colon surgery, hysterectomy, vascular surgery. | Patients less than 18 years of age; patients who were receiving antibiotics within 24 hours prior to arrival (except colon surgery patients taking oral prophylactic antibiotics); patients who were receiving antibiotics more than 24 hours prior to surgery; patients who had other procedures requiring general or spinal anesthesia that occurred within 3 days (4 days for CABG or other cardiac surgery) prior to or after the procedure of interest (during separate surgical episodes) during this hospital stay. |
| Prophylactic antibiotic selection for surgical patients - overall rate | Patients 18 years of age or older with a principal ICD-9-CM discharge diagnosis of CABG, other cardiac surgery, hip or knee arthroplasty, colon surgery, hysterectomy, vascular surgery. | Patients less than 18 years of age; patients who were receiving antibiotics within 24 hours prior to arrival (except colon surgery patients taking oral prophylactic antibiotics); patients who were receiving antibiotics more than 24 hours prior to surgery; patient did not receive prophylactic antibiotics; Patients who did not receive any antibiotics during this hospitalization. |
| Prophylactic antibiotics discontinued within 24 hours after surgery end time - Overall rate | Patients 18 years of age or older with a principal ICD-9-CM discharge diagnosis of CABG, other cardiac surgery, hip or knee arthroplasty, colon surgery, hysterectomy, vascular surgery. | Patients less than 18 years of age; patients who were receiving antibiotics within 24 hours prior to arrival (except colon surgery patients taking oral prophylactic antibiotics); patients who were receiving antibiotics more than 24 hours prior to surgery; patients who had other procedures requiring general or spinal anesthesia that occurred within 3 days (4 days for CABG or Other Cardiac Surgery) prior to or after the procedure of interest (during separate surgical episodes) during this hospital stay; patients who had a principal diagnosis suggestive of preoperative infectious diseases. |
| Cardiac surgery patients with controlled 6 a.m. postoperative blood glucose | Patients 18 years of age or older with a principal ICD-9-CM discharge diagnosis of CABG or other cardiac surgery. | Patients less than 18 years of age; burn and transplant patients. |
| Surgery patients with appropriate hair removal | Patients 18 years of age or older with a principal ICD-9-CM discharge diagnosis of CABG, other cardiac surgery, hip or knee arthroplasty, colon surgery, hysterectomy, vascular surger . | Patients less than 18 years of age; patients who performed their own hair removal. |
| Colorectal surgery patients with immediate postoperative normothermia | Patients 18 years of age or older with a principal ICD-9-CM discharge diagnosis of colorectal surgery. | Patients less than 18 years of age; patients who expired perioperatively; burn patients. |
| Surgery patients on beta-blocker therapy prior to admission who received a beta-blocker during the perioperative period | Patients 18 years of age or older, who were on beta-blocker therapy prior to admission, with a principal ICD-9-CM discharge diagnosis of CABG, other cardiac surgery, hip or knee arthroplasty, colon surgery, hysterectomy, vascular surgery. | Patients less than 18 years of age; patients who expired perioperatively; patients who did not receive beta-blockers due to contraindications. |
| Surgery patients with recommended venous thromboembolism prophylaxis ordered | Patients 18 years of age or older with a principal ICD-9-CM discharge diagnosis of CABG, other cardiac surgery, hip or knee arthroplasty, colon surgery, hysterectomy, vascular surgery. | Patients less than 18 years of age; patients with procedures performed entirely by laparoscope; patients whose total surgery time is less than or equal to 60 minutes; patients who stayed less than or equal to 3 calendar days postoperatively; burn patients; patients who are on warfarin prior to admission; patients with contraindications to both mechanical and pharmacological prophylaxis. |
| Surgery patients who received appropriate venous thromboembolism prophylaxis within 24 hours prior to surgery to 24 hours after surgery | Patients 18 years of age or older with a principal ICD-9-CM discharge diagnosis of CABG, other cardiac surgery, hip or knee arthroplasty, colon surgery, hysterectomy, vascular surgery. | Patients less than 18 years of age; patients with procedures performed entirely by laparoscope; patients whose total surgery time is less than or equal to 60 minutes; patients who stayed less than or equal to 3 calendar days postoperatively; burn patients; patients who are on warfarin prior to admission; patients with contraindications to both mechanical and pharmacological prophylaxis; patients who did not receive venous thromboembolism prophylaxis. |

ACE = angiotensin-converting enzyme; ARB = angiotensin receptor blocker; PCI = percutaneous coronary intervention; ICU = intensive care unit; CABG = coronary artery bypass grafting
